# Fluid management in wound care

**DOI:** 10.1308/003588412X13373405386015h

**Published:** 2012-09

**Authors:** J Ring, F Choukairi, J Davies

**Affiliations:** ^1^Salford Royal NHS Foundation Trust,UK; ^2^University Hospital of South Manchester NHS Foundation Trust,UK

Wounds come in many shapes and sizes often requiring washout and exploration. The limb is prepared and draped in the desired fashion. A large sterile x-ray C-arm cover is placed under the limb and secured proximally with a towel clip. A suction tube is placed in the lowest part. This tube can be cut and dropped into the bag when washout is completed and the bag removed, allowing further procedures or dressings to be performed. This technique keeps the patient, floor and team dry and allows hands-free fluid disposal, leaving clean, dry drapes for wound dressing and ease of transfer.

